# Supplementary material for: Role of the Sortase A in the Release of Cell-Wall Proteinase PrtS in the Growth Medium of Streptococcus thermophilus 4F44
Source: Microorganisms. 2021 Nov 18;9(11):2380. doi: 10.3390/microorganisms9112380 (PMC8623714; doi:10.3390/microorganisms9112380)
Supplement: Supplementary file 1 [file microorganisms-09-02380-s001.zip › microorganisms-1427343-supplementary.pdf]

Supplementary Materials

The docking of the sorting pattern LPNTG against Sp-SrtA$_{SF370\Delta86}$ led to 100 poses. Four of them were rejected as the LPNTG ligand was outside the catalytic cavity. In the 96 other poses, the LPNTG ligand was within the cavity. To understand the direction that the LPNTG ligand adopts in the catalytic cavity, we divided this cavity into two parts, designated cavity 1 and cavity 2 (Fig. 3). In 24 poses, the LP residues of the ligand were located in cavity 1 that contains the catalytic residue Arg216 whereas the TG residues were orientated towards the catalytic Cys208 (cavity 2) (Fig. 3). Contrarily, in other 22 poses, the ligand adopted the opposite orientation. In the other remaining poses, the ligand was curved in cavity 1. Only the 24 poses that had the LP residues in cavity 1 and the TG residues in cavity 2 were selected for further analysis. More precisely, the distance between the S atom of the Cys208 of Sp-SrtA$_{SF370\Delta86}$ and the alpha carbon of the threonine residue of the ligand (d[Cα(Thr)-SH(Cys208)]) was measured in order to evaluate the propensity to make a nucleophilic attack, as described in the catalytic mechanism of the sortase. Eight of the 24 poses had a distance d[Cα(Thr)-SH(Cys208)] below 4 Å. This distance was between 4 Å and 5 Å in 5 other poses. In 11 poses, the distance was higher than 5 Å. The interactions between the ligand and the sortase were analyzed using 2D models (Fig. A2). LP residues of the ligand were shown to interact with hydrophobic residues located in cavity 1 (Met125, Val186, Ala187, Val191, Val193, Ile194, Ile218). Electrostatic interactions were also found with the hydrophilic residues Arg190 and Arg216. TG residues interacted with residues of the cavity 2 through electrostatic and hydrophobic interactions (Ile211, Asp210, Val206, His143, Thr207, Ser141, His142, Cys208, Phe145, Leu113, Ala213 and Leu118). In such binding modes, the localization of the TG pattern was expected to permit the nucleophilic attack by the residue Cys208. The presence of the residues His143 and His142 could help proton transfer. All these results were consistent with those obtained by Race et al. [24], and thus validate our modeling approach.

A similar protocol was applied for the docking of the ligand LPNTG against St-SrtA$_{4F44\Delta90}$ and St-SrtA$_{LMD-9\Delta90}$. For the two systems, 94 and 96 poses were obtained respectively with the LPNTG pattern within the catalytic cavity. As described with Sp-SrtA$_{SF370\Delta86}$, the ligand adopted different orientations towards both catalytic cavities. In the case of the sortase St-SrtA$_{4F44\Delta90}$ 6 poses were obtained with the distance d[Cα(Thr)-SH(Cys208)] below 4 Å while 5 poses were found in the case of the sortase St-SrtA$_{LMD-9\Delta90}$. Interactions between the LPNTG motif and the residues constituting the catalytic cavity of St-SrtA$_{4F44\Delta90}$ were identical to those observed in the case of Sp-SrtA$_{SF370\Delta86}$. Nevertheless, 4 additional residues (Ala140, Gly114, Pro188 and Asn115) interacting with the ligand may be mentioned (Fig. A2). In the case of St-SrtA$_{LMD-9\Delta90}$, similar trends were observed. However, in this case only 18 residues constituting the cavity interacted with the ligand versus 21 for Sp-SrtA$_{SF370\Delta86}$ and 25 for St-SrtA$_{4F44\Delta90}$. Main residues interacting with the ligand in St- St-SrtA$_{LMD-9\Delta90}$:LPNTG complexes were also found in St-SrtA$_{4F44\Delta90}$:LPNTG and Sp-SrtA$_{SF370\Delta86}$:LPNTG complexes (Fig. A2). The residues Val186, Val193, Val194, Val206, Ile218 which are thought to be important for the binding of the LPNTG pattern within the catalytic cavity of the sortase A were not identified as interacting residues in St-SrtA$_{LMD-9\Delta90}$:LPNTG complexes. Therefore, these analyses did not show any significant difference concerning the binding of the LPNTG motif to Sp-SrtA$_{SF370\Delta86}$:LPNTG, St-SrtA$_{LMD-9\Delta90}$:LPNTG and St-SrtA$_{4F44\Delta90}$:LPNTG complexes.

Due to the large number of poses obtained with wrong orientation of the LPNTG motif or its folding within the catalytic cavity, further simulations were run with a larger ligand including four additional residues. Moreover, the C-terminal and the N-terminal extremities of the ligand were blocked with an acetyl and a methyl group, respectively [41]. The obtained ligand Ace-QLPNTGEND-Nme was docked against the three systems Sp-SrtA$_{SF370\Delta86}$, St-SrtA$_{4F44\Delta90}$ and St-SrtA$_{LMD-9\Delta90}$. Forty-five poses were automatically retained for the target Sp-

$SrtA_{SF370\Delta86}$ versus 34 and 35 poses for the targets St-$SrtA_{4F44\Delta90}$ and St-$SrtA_{LMD\text{-}9\Delta90}$. The complexes were analyzed based on the same criteria than previously described (orientation of the ligand towards the catalytic cavity and proximity of the TG residues to the catalytic cystein). In the case of Sp-$SrtA_{SF370\Delta86}$, the ligand was recurved in cavity 1 in 18 poses (binding mode 1). In 23 poses, the moiety Ace-QLPNT was positioned within cavity 1 whereas the rest of the ligand was outside the catalytic cavity (binding mode 2). In 4 poses, the moiety Ace-QLPNT was localized in cavity 1 whereas the other moiety of the ligand was located within cavity 2 (binding mode 3). Among them, two poses had the distance d[Cα(Thr)-SH(Cys208)] below 5 Å (3,31 Å and 4.32 Å). In the case of St-$SrtA_{4F44\Delta90}$, 12 poses adopted the binding mode 1; thirteen poses were similar to binding mode 2; three poses adopted the binding mode 3. In the rest of the poses, the moieties Ace-QLPNT and GEND-Nme were oriented towards cavity 2 and cavity 1, respectively. Similar trends were obtained with the system St-$SrtA_{LMD\text{-}9\Delta90}$. The interactions between the catalytic cavity and the ligand were similar for the three systems and similar to those previously described for the ligand LPNTG. Moreover, the superimposition of the different complexes (Fig. A3) showed no significant difference in the orientation and the conformation of the pattern LPNTG within the cavity.

Table S1 Primers and resulting PCR or Overlapping PCR products used for mutant construction.

Primer	Figure annotation	Sequence (5'→3')	Target	DNA matrix	PCR product (bp)	AT°C in PCR	OL PCR P. L. (bp)	AT°C in OL PCR	Mutant construction
*UpprtMF	Up-*X*F	AGCCTTGGTTGAAAAAGGTG	*UpprtM*	LMD-9 gDNA	987	55.7	2556	52.2	LMD-$9_{\Delta prtM}$
UpprtMR	Up-*X*R	CATTTTATCTACTCCTTAAATTGTC							
DownprtMF	Down-*X*F	ATCATAAGATAAAGTTGATAATGTTG	*DownprtM*	LMD-9 gDNA	834	55.7			
*DownprtMR	Down-*X*R	AAGGACCTGCACCGATTTC							
prtMeryF	*X-ery*F	*GACAATTTAAGGAGTAGATAAA*ATGAACAAAAATATAAAATATTCTC	*ery*	pG+host9	786	*58.2*			
prtMeryR	*X-ery*R	*CAACATTATCAACTTTATCTTATGATTTA*TTTCCTCCCGTTAAATAATAG							
*UpprtSF	Up-*X*F	ACTCCGAGGTAGCAGACCAA	*UpprtS*	LMD-9 gDNA	821	56.2	7530	50	LMD-$9_{prtS4F44}$
prtSUpR	Up-*X*R	TGCAGCTACCGATGGTGC							
SSprtSF	Up-*prtS*-4F44F	GCACCATCGGTAGCTGCA	*prtS*$_{4F44}$	4F44 gDNA	4671	56.2			
prtSR	*prtS*-4F44R	TTATACTTCTTCCTTGTGACGTCGTAT							
prtSSpecF	*X-spec*F	*ATACGACGTCACAAGGAAGAAGTATAA*CGTAACGTGACTGGCAAGAG	*spec*	pSET4s	1188	*60.2*			
prtSSpecR	*X-spec*R	*CACAATGATTAAGCGGTTTTATT*ATCGCTACGATAACGCCTGTTT							
DownprtSF	Down-*X*F	ATAATAAAACCGCTTAATCATTGTG	*DownprtS*	LMD-9 gDNA	920	56.2			
*DownprtSR	Down-*X*R	TTTCTTGATGACGCAACCAC							
*UpgyraseF	Up-*X*F	GACAGGTGTGAACGATGACG	*UpsrtA*	LMD-9 gDNA	870	56.2	2408 (*UpsrtA-ery-DownsrtA*) / 3571 (*UpsrtA-srtA*$_{4F44}$*-spec-DownsrtA*) / 3571 (*UpsrtA-srtA*$_{LMD-9}$*-spec-DownsrtA*)	50	LMD-$9_{\Delta srtA}$ LMD-$9_{srtALMD-9}$ LMD-$9_{srtA4F44}$
UpgyraseR	Up-*X*R	TAGTTATCACCACTTTATTGATCTTC							
gyr-eryF	*X-ery*F	*GAAGATCAATAAAGTGGTGATAACTAA*TGAACAAAAATATAAAATATTC	*ery*	pG+host9	781	56.2			
lyase-eryR	*X-ery*R	*GACCTTACGGCCTTTTC*TTATTTCCTCCCGTTAAATAATAG							
DownlyaseF	Down-*X*F	TAAGAAAAGGCCGTAAGGTC	*DownsrtA*	LMD-9 gDNA	803	56.2			
*DownlyaseR	Down-*X*R	ATTTGCATAGGCCGTAGTC							
Up-srtA-LMD9F	Up-*X*-LMD9F	GAAGATCAATAAAGTGGTGATAACTA	*srtA*$_{4F44}$ / *srtA*$_{LMD-9}$	4F44 gDNA / LMD-9 gDNA	788	60.2			
srtA-LMD9R	*X*-LMD9R	TTAACTTTGAAATTGATTGTATGACT							
SpecF	*X-spec*F	*GTCATACAATCAATTTCAAAGT*TAACGTGACTGGCAAGAG	*Spec*	pSET4s	1176	60.2			
SpecR1	*X-spec*R	*GACCTTACGGCCTTTTCTTA*CGCTACGATAACGCCTGTTT							

Table S1 (Continued)

Primer	Figure annotation	Sequence (5'→3')	Target	DNA matrix	PCR product (bp)	AT°C in PCR	OL PCR P. L. (bp)	AT°C in OL PCR	Mutant construction
*Up*gyrase*F	Up-*srtA*F	GACAGGTGTGAACGATGACG	Up*srtA*+*srtA*$_{LMD\text{-}9}$	LMD-9$_{srtALMD\text{-}9}$ *gDNA*	1545	56.2	3571 (Up*srtA*+*srtA*$_{LMD\text{-}9}$ - *srtALMD*$_{\text{-}9}$ + *spec*$_{srtA}$ + Down *srtA*)	50	LMD-9$_{srtA\text{:Ile218}\rightarrow\text{Val218}}$
srtA$_{V218R}$	*srtA*$_{V218R}$	CCCTTTGACAACTGTACGCTG							
srtA$_{V218F}$	*srtA*$_{V218F}$	CAGCGTACAGTTGTCAAAGGG	*srtA*$_{LMD\text{-}9}$ + *spec*$_{srtA}$ + Down *srtA*		2026	56.2			
*Down*lyase*R	Down-*srtA*R	ATTTGCATAGGCCGTAGTC							

AT: annealing temperature; LMD-9/4F44 gDNA: genomic DNA of the strain LMD-9 or 4F44; *primers used to amplify recombinant DNA fragments by overlapping (OL) PCR; Italicized nucleotides correspond to homologous tails; *UpprtM*: fragment located upstream of the *prtM* gene; *DownprtM*: fragment located downstream of the *prtM* gene; *ery*: erythromycin resistance gene; *UpprtS*: fragment located upstream of the *prtS* gene; *prtS*$_{4F44}$: *prtS* allele of the strain 4F44; *spec*: spectinomycin resistance gene; *DownprtS:* fragment located downstream of the *prtS* gene; *UpsrtA*: fragment located upstream of the *srtA* gene; *DownsrtA*: fragment located downstream of the *srtA* gene; *srtA*$_{4F44}$: *srtA* allele of the strain 4F44; *srtA*$_{LMD\text{-}9}$: *srtA* allele of the strain LMD-9.

Table S2 Primers used for the sequencing of PPIases genes.

Primer couple	Sequences (5'→3')	Gene
**ppiA1forLMD-9 \ ppiA1rev**	TTGACAATGCACCAATTGAGA \ TAGGAGCAAGCGTAGGGAAG	*ppiA*
**ppiA2forLMD-9 \ ppiA2rev**	CTTGACAGGGAGCTATTCACG \ CTTTAATGACTTCGACTGAATTAACA	
**ppiA3for \ ppiA3rev**	GAAAGGTTTCGCTGTTGAGC \ CCGCTTATCGGGAATATTGT	
**pplB1for \ pplB1rev**	AAGGATGGTTACGCCCAAAT \ CAGGTTCTAGGTCCAGAATTTCC	*pplB*
**pplB2for \ pplB2rev**	TTTTGCTTTTTAGCTTTAAGAAATGA \ TGGCTTTAAGTTCTGGTGTGG	
**pplB3for \ pplB3rev**	CGTTCAGAATTGATTGATGATG \ AAGCTTCTGCAATTGGTGCT	
**pplB4for \ pplB4rev**	TCAAGCTTTTCCCTGACCAT \ ACGCTTCTGCCAAAGGTTTA	
**pplB5for \ pplB5rev**	CGCTTGAGGAAGTTGTTATTGA \ AGGACCATTTCGTTTGCACT	
**prtM1for \ prtM1rev**	CCTGATGCTCAGATTACGACAG \ GAGGGCAATGCTTTCCTCTT	*prtM*
**prtM2for \ prtM2rev**	TTGCCAAAGACATCATGGAG \ TGTGCAGCTTGGTTAACAGC	
**prtM3for \ prtM3rev**	TGGTGCTGGTGCTATGTATG \ TGAAGCTCCAGAACCAGTCA	
**prtM4for \ prtM4rev**	CGAAAGTTGCTAAGGATAACAGC \ GGGTCGTTAACTGGAACCAA	
**prtM6for \ prtM6rev**	TGCTACAGGATTTGCAGCAG \ CTTGGCAAAGCCAATCATTT	
**prtM7for \ prtM7rev**	GCTGAACTGGAGGTCTTTGC \ AATGCCCTTTACCTGTTCCA	
**tig1for \ tig1rev**	GAATCCTTGAGCTCCTAGACCA \ GGTTAAATACGGCACGAGGA	*tig*
**tig2for \ tig2rev**	GCATTAGGCTTTCTTCGAAAT \ CCTGCAAGATCAGTAGCTTGA	
**tig3for \ tig3rev**	TGCAGAAGTTGTTACAAAACCA \ TCAGCTGCAAGGTCATTGAT	
**tig4for \ tig4rev**	AAATTGCATTTGATGATGCTG \ AAGCCCCATTTGTTATCACC	
**tig5for \ tig5rev**	TATGTCTAAAATCAAGTTTCTGTTGAG \ CGTTTTGGTGCATTTTCATC	

PCR reactions were carried out at an annealing temperature of 55°C.

Table S3 Primers used for the verification of mutant sequences.

Primers couple's	Sequences (5'→3')	sequenced mutant
**gyrA2rev \ gyrA2for**	CGTGGAGGTCGTGGAGTTC \ TGTCTTAATACCTTTACCACCA	
**gyrA1rev \ ERY1 R**	CAGTGTGCGTTTCAAAGAGGA \ TTGAGTGTGCAAGAGCAACC	
**Ery1F \ Lyase1R**	AAAGGGCATTTAACGACGAA \ TAATCAGAAACAATGATAGCGACA	
**Sort3F \ Spec2R**	TCAAAGGGAACTTGGAATCG \ AGCAAGAAATGGTACCGTGG	**LMD-9$_{\Delta srtA}$**
**gyrA1rev \ Sort2forR**	CAGTGTGCGTTTCAAAGAGGA \ TATCAGAAGCCCCAGCGATA	**LMD-9$_{srtA\text{LMD-9}}$**
**Sort1rev \ Spec1R**	CCCAGATGGGTTCTCAAAAA \ TCCTCCTCACTATTTTGATTAGTACCT	**LMD-9$_{srtA\text{4F44}}$**
**Spec1F \ Lyase1R**	TCAAAATAGTGAGGAGGATATATTTGA \	**LMD-9$_{srtA\text{:Ile218→Val218}}$**
**Spec2F \ Lyase2R**	GGGAGAGAATTTTGTTAGCAGTTC \ CCTAAAAGATGACCGAGTGTGA	
**Lyase1F \ LyasechrLMD-9R**	CCAACCTATTCGCAAGGACA \GCAAAGTTCACCAAAAGATTGA	
**UpUpF \ HEmS02R**	TACCAGCCAAATTCATCACG \ AAACTGCACCCAAAAGAACAG	
**Style 1 \ Style 2**	CCAACAACAAGGCACTCATTA \ GTTGAGTTGTTTCTACTTCTGT	
**Style 3 \ Style 4**	ACTAAGGTGGAAGCAACTGTT \ ATTGTCCAGCGACTGGTTGT	
**PrtFor \ PrtRev**	TACGGTGAATGGTTTAACG \ AATTACTTTACTACCAACCG	
**Style 5 \ Style 6**	CAGCTGGTAATGATGGAACAT \ CCAACTTGAAAAATCTGAAAGAA	
**Style 7 \ Style 8**	CAATTCCATCTGTCTTCATTC \ CCGTTGTTAATTGTGTTGAGT	
**Style 9 \ Style 10**	GTTTATATTTGACTGGCGAAG \ GTCTTGATCGCAGAGTCAG	
**Style 11 \ Style 12**	GAACCTGTTACAGCACAGC \ GCTGGACGAGGGTTAAATG	
**Style 13 \ Style 14**	GTTCCAGGTGCAGCAGTACA \ ACCAAATGGTAGCTTAAGAACG	**LMD-9$_{prtS\text{4F44}}$**
**Style 15 \ Style 16**	TCAGTCACCGATGAAACAGG \ ATAGGCAGTTTGAAGTTCTGG	
**Style 17 \ Style 18**	ACTTGCAAACCAGTCAAATGTTA \ CGCTGCTTCAACTGCCTGG	
**Style 19 \ Style 20**	GCAGCGAAAGCCAACTTAGA \ TTCCTTGTGACGTCGTATGC	
**Style 20-1 \ Spec2R**	ATTCAGAGCATGTACCGATAG \ CACAATGATTAAGCGGTTTTATTAT	
**Spec1F \ HEMS17 R**	TCAAAATAGTGAGGAGGATATATTTGA \ TGATCTGAGAACATAAGGGTC	
**Spec2F \ Style 20-2**	GGGAGAGAATTTTGTTAGCAGTTC \ GGTGTTGATGGGCTAGAGGA	
**Style 21F \ PrtSDnDnR**	GGTGTTGATGGGCTAGAGGA \ ACGACGATAACCAAGAATGC	
**prtM6F \ prtM6R**	TGCTACAGGATTTGCAGCAG \ CTTGGCAAAGCCAATCATTT	
**prtM7F \ prtM7R**	GCTGAACTGGAGGTCTTTGC \ AATGCCCTTTACCTGTTCCA	
**prtM2F \ ery 1 R**	TTGCCAAAGACATCATGGAG \ TTGAGTGTGCAAGAGCAACC	**LMD-9$_{\Delta prtM}$**
**ery 1 F \ prtM9R**	AAAGGGCATTTAACGACGAA \ CGACCGCCACTTTTCTGTAT	
**DownprtMF \ DownprtMR**	ATCATAAGATAAAGTTGATAATGTTG \ AAGGACCTGCACCGATTTC	
**prtM8F \ prtM8R**	**CCACACGATGTTTGAAATGC \ CGGAGAAGACGACGAAGAAC**	

PCR reactions were carried out at an annealing temperature of 55°C
